# Supplementary material for: Dynamic Landscape of Extracellular Vesicle-Associated Proteins Is Related to Treatment Response of Patients with Metastatic Breast Cancer
Source: Membranes (Basel). 2021 Nov 16;11(11):880. doi: 10.3390/membranes11110880 (PMC8619728; doi:10.3390/membranes11110880)

Figure S1. Full and uncropped western blot results.

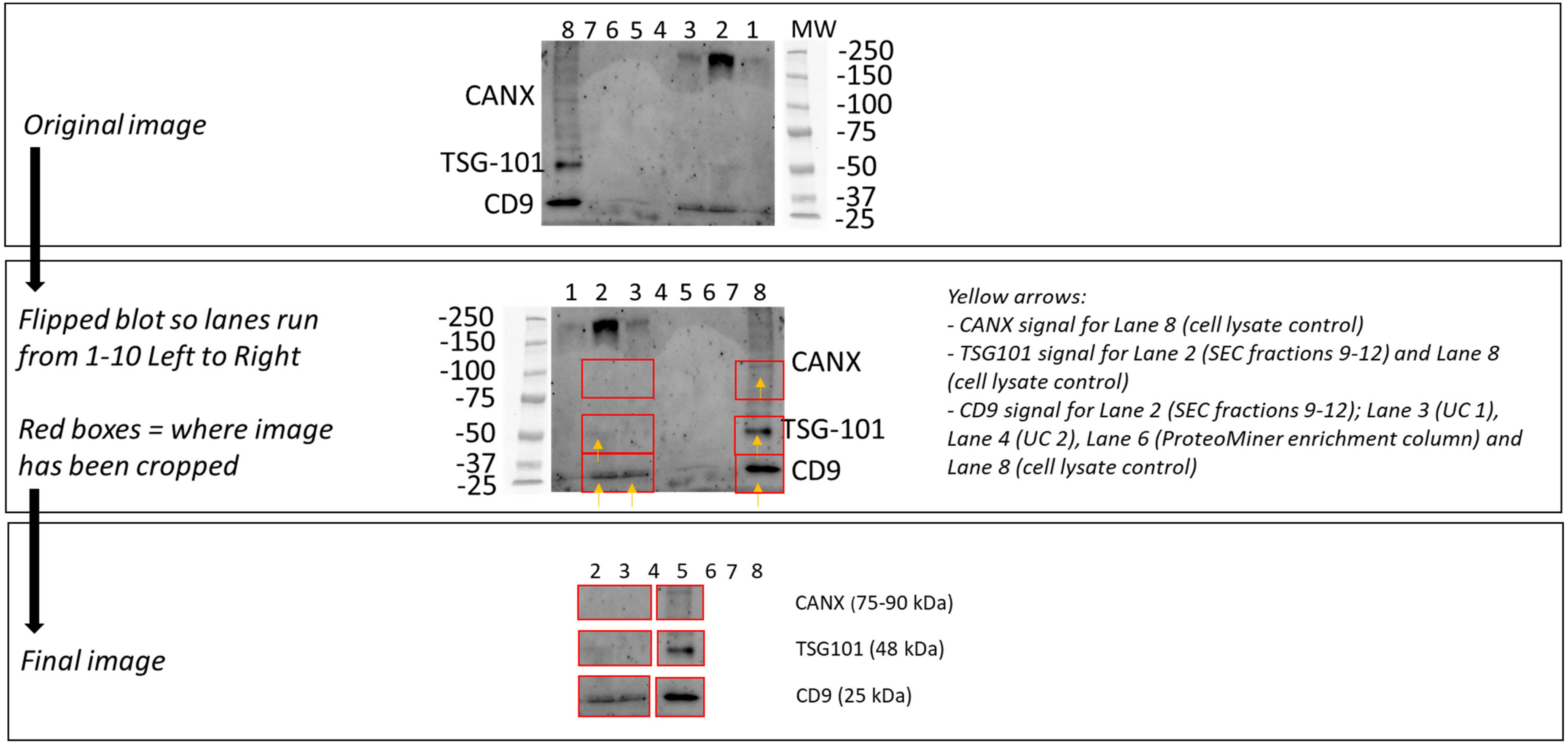

Figure S2. String analysis revealed the presence of two clusters within 23 EV associated proteins that were uniquely up-regulated in the non-responder.

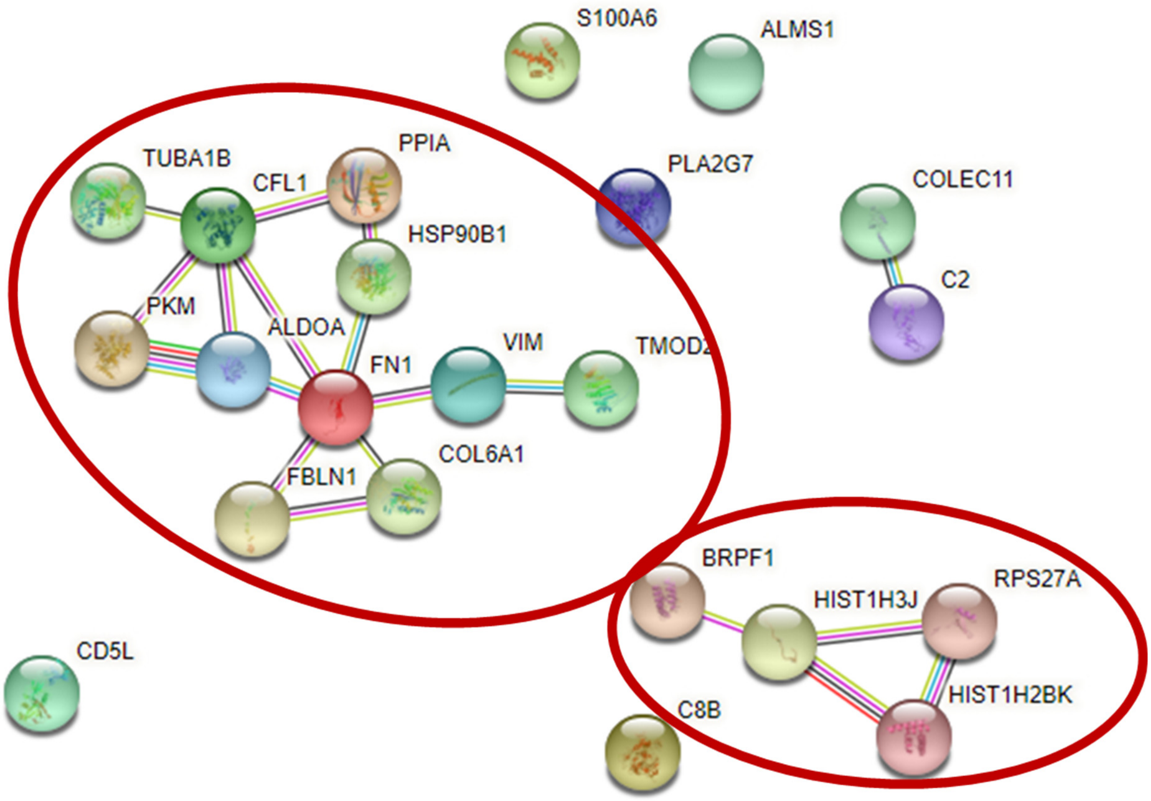

Supplement: Supplementary file 1 [file membranes-11-00880-s001.zip › revised_Supplementary Figures.pdf]
