# Supplementary material for: Dynamic Landscape of Extracellular Vesicle-Associated Proteins Is Related to Treatment Response of Patients with Metastatic Breast Cancer
Source: Membranes (Basel). 2021 Nov 16;11(11):880. doi: 10.3390/membranes11110880 (PMC8619728; doi:10.3390/membranes11110880)
Supplement: Supplementary file 1 [file membranes-11-00880-s001.zip › Supplementary Data Table S5.pdf]

Supplementary Data Table S5. Summary of candidate EV-associated proteins have a potential association with breast cancer aggression/metastasis/invasion and survival.

			RNA expression, TCGA/Protein Atlas			Protein expression, Tang 2018		
	Protein ID	Name	Type	p value	Survival	p value	low	high
Increased in the non-responder	P23528	Cofilin-1	T	<1E-12	NS	0.017	100	27
	P14618	Pyruvate kinase PKM	-	-	-	0.034	100	36
	P04075	Fructose-bisphosphate aldolase A	T	<1E-12	NS	0.044	97	17
	P68363	Tubulin alpha-1B chain	T	1.62E-12	0.00035	NS	-	-
	P62937	Peptidyl-prolyl cis-trans isomerase A	T	<1E-12	NS	0.032	100	36
	P02751	Fibronectin	T	<1E-12	NS	0.0046	36	15
	P14625	Endoplasmin	T	1.62E-12	NS	NS	-	-
	P08670	Vimentin	N	1.62E-12	NS	NS	-	-
	Q9NZR1	Tropomodulin-2	N	<1E-12	NS	0.0068	97	48
	P12109	Collagen alpha-1(VI) chain	T	<1E-12	NS	NS	-	-
	P55201	Peregrin	-	NS	NS	NS	-	-
	P68431	Histone H3.1	T	3.70E-05	NS	0.015	97	23
	O60814	Histone H2B type 1-K	T	1.62E-12	NS	NS	-	-
	P62979	Ubiquitin-40S ribosomal protein S27a	N	<1E-12	NS	NS	-	-
	P06681	Complement C2	T	2.27E-05	NS	0.0013	-	-
	Q13093	Platelet-activating factor acetylhydrolase	T	9.13E-02	0.033	-	-	-
	Q8TCU4	Alstrom syndrome protein 1	T	3.57E-02	NS	0.045	41	24
	P07358	Complement component C8 beta chain	-	NS	NS	NS	-	-
	P06703	Protein S100-A6	N	1.77E-05	NS	0.025	10	39
	P23142	Fibulin-1	N	2.18E-11	NS	0.0052	97	16
	Q9BWP8	Collectin-11	N	1.51E-06	NS	-	-	-
	P03952	Plasma kallikrein	N	1.33E-07	NS	0.025	23	48
	O43866	CD5 antigen-like	-	NS	NS	-	-	-
Decreased in the non-responder	Q8WZ42	Titin	-	NS	NS	0.037	39	13
	P02730	Band 3 anion transport protein	N	1.23E-04	NS	NS	-	-
	P08697	Alpha-2-antiplasmin	N	6.29E-05	NS	NS	-	-
	Q96H78	Solute carrier family 25 member 44	T	1.62E-12	NS	NS	-	-
	P27169	Serum paraoxonase/arylesterase 1	N	4.77E-02	NS	NS	-	-
	Q96Q89	Kinesin-like protein KIF20B	T	1.62E-12	NS	NS	-	-
	P20851	C4b-binding protein beta chain	-	NS	NS	-	-	-
Increased in the responder	P04406	Glyceraldehyde-3-phosphate dehydrogenase	T	<1E-12	NS	0.015	97	17
	Q6FI13	Histone H2A type 2-A	-	-	-	0.0089	100	27
	Q13201	Multimerin-1	N	<1E-12	NS	NS	-	-
	P32119	Peroxiredoxin-2	T	<1E-12	NS	0.029	70	23
	P11277	Spectrin beta chain, erythrocytic	N	4.44E-03	NS	NS	-	-
